# Evaluating the Population-Based Usage and Benefit of Digitally Collected Patient-Reported Outcomes and Experiences in Patients With Chronic Diseases: The PROMchronic Study Protocol

**DOI:** 10.2196/56487

**Published:** 2024-08-05

**Authors:** Janis Nikkhah, Viktoria Steinbeck, Thomas G Grobe, Thorben Breitkreuz, Christoph Pross, Reinhard Busse

**Affiliations:** 1 Department of Healthcare Management School of Economics and Management Technical University Berlin Berlin Germany; 2 aQua Institute Göttingen Germany

**Keywords:** health care policy, PROM, PREM, PRO feedback, health behavior, chronic disease, asthma, COPD, diabetes, coronary artery disease, value-based health care, quality of care, artery disease, chronic, self-management, effectiveness, Germany, cohort study, pulmonary disease, utilization

## Abstract

**Background:**

Chronic diseases are associated with a high disease burden. Under- and overprovision of care as well as quality variation between health care providers persists, while current quality indicators rarely capture the patients’ perspective. Capturing patient-reported outcome measures (PROMs) as well as patient-reported experience measures (PREMs) is becoming more and more important to identify gaps in care provision, prioritize services most valuable to patients, and aid patients' self-management.

**Objective:**

This study aims to measure the potential benefits and effectiveness of using electronic patient-reported outcome measures (ePROMs) and electronic patient-reported experience measures in a structured and population-based manner to enhance health care for chronic disease patients in Germany.

**Methods:**

This prospective cohort study aims to evaluate the potential benefits of PROM usage in patients with chronic diseases. We evaluate whether (1) digitally collected PROMs and PREMs can be used for health system performance assessment by generating a representative response of chronically diseased individuals with asthma, chronic obstructive pulmonary disease, diabetes, and coronary artery disease across Germany, and (2) based on the PROMs and PREMs, low-value care can be identified. As patient-reported outcomes (PROs) are rarely presented back to patients, (3) this study also examines patients’ reactions to their PROM scores in the form of digital PRO feedback. For these purposes, randomly selected patients from a nationwide German insurer are digitally surveyed with generic and disease-specific PROMs and PREMs, as well as additional questions on their health-related behavior, 4 times over 1 year. Individual PRO feedback is presented back to patients longitudinally and compared to a peer group after each survey period. Patient-reported data is linked with health insurance data. Response rates, changes in health and experience outcomes over time, self-reported changes in health behavior, and health care system usage will be analyzed.

**Results:**

The PROMchronic study explores the usage of PROMs in patients with chronic diseases. Data collection began in October 2023, after the initial invitation letter. All the 200,000 potential patients have been invited to participate in the study. Data have not yet been analyzed. Publication of the interim results is planned for the autumn of 2024, and the results are planned to be published in 2025.

**Conclusions:**

We aim to fill the research gap on the population-based usage of PROMs and PREMs in patients with chronic diseases and add to the current understanding of PROM data-sharing with patients. The study’s results can thereby inform whether a health care system-wide approach to collecting PROMs and PREMs can be used to identify low-value care, assess quality variation within and across chronic conditions, and determine whether PRO feedback is helpful and associated with any changes in patients’ health behaviors.

**Trial Registration:**

German Clinical Trials Register DRKS00031656; https://drks.de/search/en/trial/DRKS00031656

**International Registered Report Identifier (IRRID):**

DERR1-10.2196/56487

## Introduction

### Background and Rationale

Globally, 1 in 3 persons and up to 60% in industrialized countries are living with at least 1 chronic disease [1-3]. Furthermore, this share is continuously increasing across the globe due to demographic change and consumption patterns [3,4]. Chronic diseases, including conditions such as diabetes, cardiovascular diseases, and chronic obstructive pulmonary disease (COPD), are long-lasting conditions that can significantly impact a patient's quality of life and are a key driver of escalating health costs in both developed and developing economies [5]. Diagnosing and treating chronic conditions comes with substantial uncertainties for patients, families, and caregivers [6]. The high complexity of chronic care, for instance, raises uncertainty regarding whether patients with chronic diseases will respond to a selected treatment in the way a priori expected and, in the event they do, whether it is the most efficient one [7].

Patient-reported outcome measures (PROMs) and patient-reported experience measures (PREMs) have been used for over 20 years, but have become increasingly widespread and accepted only in recent years [8], as reflected by their increasing usage in oncology [9-11] and orthopedics [12-14]. The use of PROMs in monitoring interventions has, for instance, shown benefits in terms of increased survival rates, lower hospitalizations, improved health-related quality of life in oncology and reduced fatigue, and improved health-related quality of life in orthopedics [15,16]. PROMs are questionnaires that allow valuable insights into the patient’s perspective of living with a (chronic) disease as they reflect the patient’s own perception of their health status [17,18]. PREMs are questionnaires used to gather information from patients about their personal experiences and perceptions of the care and services they have received [19]. With electronic PROMs (ePROMs) and electronic PREMs (ePREMs) being reported and used more frequently in the last few years [20,21], usage for population health purposes appears more in reach but has rarely been examined [22].

PROMs and PREMs might have care improvement potential in the field of chronic diseases due to their information value for patient empowerment, including self-management and shared decision-making, which could influence treatment adherence and lifestyle choices [23]. However, PROMs and PREMs have rarely been used in routine chronic care due to the unspecified timeline and the complexity of implementation [24]. Due to the high relevance of this topic, the OECD (Organisation for Economic Co-operation and Development) PaRIS (Patient-Reported Indicator Surveys) initiative focuses on collecting and analyzing PROMs in patients with chronic diseases across 18 countries, starting their trial phase in 2023. The initiative does not cover Germany [25]. The present study, “PROMchronic,” aims to fill the research gap by evaluating the usage of ePROMs and ePREMs in the German chronic disease population for 4 selected chronic disease profiles.

Furthermore, providing patients with information on their health status based on PROMs in reference to a comparable peer group is rarely done [26,27]. Yet, specific information about patients’ health status is crucial because it can help patients better understand their health conditions and enable decision-making and overall patient empowerment. When patients are well-informed about their health, they are more likely to adhere to treatment plans and achieve better health outcomes [28]. Patients can use this information together with their health care providers to set realistic goals for their treatment, monitor their progress, and identify areas where additional support is needed.

### Objectives

Our study aims to evaluate the potential benefits of the structured and population-based use of ePROMs and ePREMs to improve care for chronically ill patients in Germany. First, we evaluated whether and how representative the response to the digitally collected questionnaires is and thereby investigate PROMs as a tool for health system performance assessment (HSPA). Second, we assessed if low-value care elements can be identified in today’s care for chronically ill patients across Germany. Third, we analyzed patients’ understanding of and reactions to individualized patient-reported outcome (PRO) feedback. To achieve these objectives, this study aims to answer 3 overarching research questions and their subquestions (Textbox 1).

**Overarching research questions and their sub-questions.**

**Can electronic patient-reported outcome measures (ePROMs) and electronic patient-reported experience measures (ePREMs) be used in patients with chronic diseases for quality measurement at the health system level (eg, for health system performance assessment [HSPA])?**

- How representative are response rates in patients with chronic diseases via digital surveys in terms of gender, age, and diagnoses?
- How do response rates and willingness to respond multiple times vary over time by age, gender, indication, city or state, health system use (frequent versus infrequent users), web- or app-based surveys, and Disease Management Program (DMP) participation?

**To what extent can value-of-care variation be identified from ePROMs and ePREMs surveys?**

- What is the share of suspected low-value care and suspected high-value care in Germany, per indication and in subgroups?
- How do ePROM and ePREM results and health indicators of health insurance data differ according to age, gender, indication, city or state, health care system usage (changes in frequency of outpatient practitioner attendance, prescriptions, hospitalizations, etc), and DMP participation?
- Can ePROMs and ePREMs function as an early warning signal for deteriorating chronic conditions or adverse events such as hospital admission?

**What are the benefits or drawbacks of patient-reported outcome (PRO) feedback (outcome reports) sent to patients?**

- Are the PRO outcome reports understandable to patients?
- Can PRO feedback function as a positive nudge (be associated with positive behavioral changes, eg, healthier or more active lifestyle, more active participation in medical treatment, actively approaching treating physicians in the office setting)?
- Are there any negative emotional reactions when receiving PRO feedback that shows values worse than those of a comparable group?

## Methods

### Trial Design

**Figure 1:**
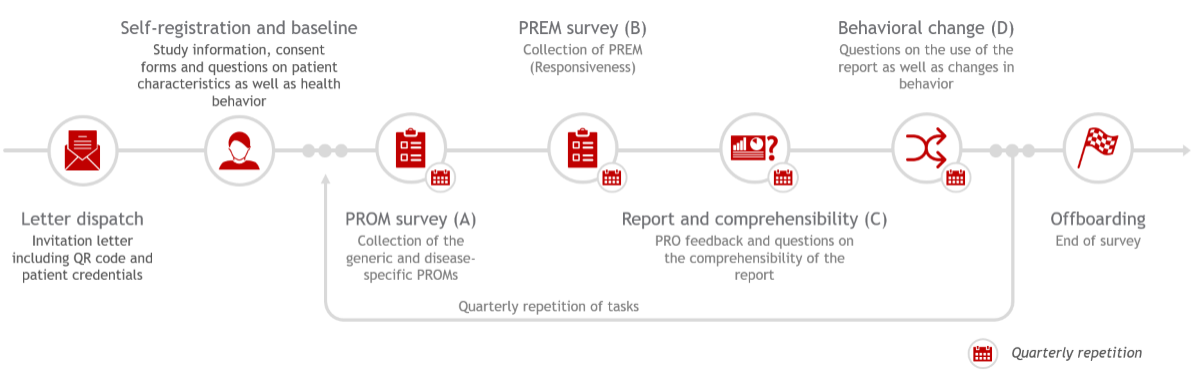
Study design: patient pathways including timeline and tasks. PREM: patient-reported experience measure; PRO: patient-reported outcome; PROM: patient-reported outcome measure.

This is an observational prospective cohort study covering the 4 chronic diseases: asthma, COPD, diabetes types 1 and 2, and coronary artery disease (CAD). As shown in Figure 1, the first point of contact will be a letter from the insurer to insured patients selected for their chronic disease. A patient can then sign up digitally for the study via a QR code or web-based link. For each of the different diseases, a distinct patient pathway is automatically assigned, with a registration process followed by some background questions and the respective generic and disease-specific PROMs and PREMs. The study covers a timeframe of 1 year, split into 4 time intervals with further information and reoccurring questionnaires. The time intervals are split into smaller tasks to facilitate the answering process for participants. The first interval includes self-registration and a baseline assessment of health behavior. A few weeks after responding to the first set of questions (Figures 1A and 1B), patients will receive a report with their individual PROM scores compared to a peer group and will be surveyed on the comprehensibility of the PRO report (Figure 1C). From the second interval onwards, patients will also receive their individual PRO results longitudinally. Lastly, following the individual PRO feedback report, participants will be surveyed regarding their health behavior (Figure 1D). After 1 year, the primary data collection process is finished, and patient-reported data will be merged with health insurance data. Further explanations of subgroup definitions can be found in the Statistical Analysis Plan (Multimedia Appendix 1).

### Study Setting

This study will recruit patients all over Germany who are insured by one of the largest statutory health insurers in Germany (BARMER). The BARMER represents more than 10% of the German population. After the identification of individuals based on their chronic disease profile, validated PROM sets are used digitally to regularly survey patients with asthma, COPD, diabetes, or CAD on their health outcomes as well as their experience with the health care system.

### Eligibility Criteria

Patients are eligible for participation in the study if they are above the age of 18 years and have at least 2 outpatient consultations in 2021 documenting a confirmed chronic disease diagnosis of 1 of the 4 focus diseases. Additionally, for type 1 diabetes patients, at least 1 insulin prescription must be documented in 2021. To contact patients and enable the linkage of health insurance data, patients must be insured by BARMER health insurance all year in 2021. Patients enrolled in a Disease Management Program (DMP) as well as patients not enrolled in any DMP are eligible. However, enrolment in more than 2 DMPs is not permitted to avoid multiple invitation letters and cross-over groups.

### Participant Timeline

Eligible participants will be participating in the study for a maximum of 4 quarters, starting with the first access to the digital questionnaires, followed by quarterly digital surveys split into several tasks (compare Figure 1). To start the survey, participants register with their study pseudonyms received in their enrollment letter and then share basic demographics. Each survey period contains 4 tasks to be completed by participants, defined as sets of questions that each will take 5-10 minutes to complete. The participants will receive individual reports on their patient-specific and peer group outcomes (PRO feedback) and will be asked about the comprehensibility of the report. Following the report, the participants receive a set of questions regarding their health-related behavior.

### Sample Size

The initial enrollment letter is sent to 200,000 insured patients, 50,000 for each of the selected chronic conditions. Diabetes type 1 and type 2 are considered jointly, as there is no differentiation in the selected PROMs. Patients with all chronic diseases are allocated to groups based on their participation in DMP (DMP or non-DMP group). Prior experiences suggest that around 30% of patients react to invitations to participate in research by their insurer [29-31]. Two-thirds of initial participants are expected to allow follow-up contacts and processing of their health insurance data, and around 40% of these patients will continue participating in all the follow-up surveys after receiving reminders for each task. Therefore, after accounting for nonparticipants and participants with no complete survey data, we expect a complete data set for 16,000 participants. However, the first research question will investigate response rates, as there is no evidence on this population yet.

BARMER Health Insurance will send out enrollment letters based on a random selection of BARMER-insured patients following the defined eligibility criteria. Matching of DMP- and non-DMP participants will be done according to the steps provided in Textbox 2.

The output is a data set containing matched pairs per indication. The first 25,000 matching pairs for each chronic condition will be selected. Diabetes type 1 and type 2 are considered jointly, with patients having diabetes type 1 selected first (approximately 6600 matched pairs are expected), followed by supplementing the remaining required matched pairs with patients having diabetes type 2.

**Matching of Disease Management Platform (DMP) and non-DMP participants.**

Stratification by:

- Gender (male or female).
- 10-year age groups (first group: 18 to younger than 30 years and last group: 90 years and older).
- Number of outpatient cases with indication diagnoses within the year 2021 (outpatient cases in less than 4 quarters, outpatient cases in all 4 quarters but less than or equal to 6 cases, outpatient cases in all 4 quarters with more than 6 cases).
- Matching DMP and non-DMP patients by strata and a generated random numbering within the strata (1:1 matching).
- Consecutive selection of randomly sorted matched pairs if both matched individuals are insured all year long.

### Recruitment

An initial enrollment letter including the study explanation, a QR code, and a link as an invitation to participate in the study will be sent to insured patients in October 2023 (Multimedia Appendix 2). The enrollment survey and, thereby, the recruitment period will be open for 2 months.

### Ethical Considerations

The study will be conducted in accordance with the Declaration of Helsinki and was approved by the Charité’s Ethic Committee, Berlin (EA2/035/23). All potentially eligible participants will be approached to offer their informed consent to participate in the study as well as to allow linkage of their insurance data for further analysis. This protocol is version 1, dated January 10, 2024. Any changes in the study design will be communicated to all project partners.

Informed consent must be provided digitally before patients can participate in the study. By signing the informed consent, patients allow contact for follow-up surveys via email and to process their survey data for academic research purposes. Additionally, patients are asked to sign a second consent form for processing and linking their insurance data to their survey data. The second consent form is optional and is not a prerequisite to participating in the study. Patients will be free to withdraw from either one of the consents without stating a reason until the anonymization of the data. Through the anonymization of the primary and secondary data before the provision of data sets to research institutions in the project, patient identification will not be possible. If the patients withdraw their consent for participation, all their data will be deleted. Consent forms can be found in Multimedia Appendix 3. Participants are not reimbursed for their participation.

### Intervention

#### Explanation for the Choice of Comparators

This prospective observation cohort study does not involve any specific therapeutic treatment. To analyze and report insights on sub-cohorts of the study population, splits for age, gender, indication, PRO feedback, and usage of the health care system (eg, DMP) are formed.

#### Intervention Description

In addition to filling out the survey questions (considered here as intervention 1), all participants will receive PRO feedback (considered here as intervention 2—see Multimedia Appendix 4). The PRO feedback will be a pdf report sent via email, which graphically (line charts) shows the patients’ individual generic and disease-specific health status in comparison to a patient-specific peer group. In addition to the visual feedback, the results will shortly be explained on the same page. The description will cover whether patient-individual PRO scores are better or worse compared to the peer group, and if worse, the patients' health status might be still good and can be discussed with the patients' physician. Additional details on the scores and sub-scores are shared with the patients on the subsequent pages of the report. Peer groups are classified according to their disease, gender, and age group (18-45 years, 45-65 years, 65-75 years, and 75 years and above). The report refers to a patient’s routine care physician as the main contact to discuss the results or if there are questions.

#### Criteria for Discontinuing or Modifying Allocated Interventions

Participants may withdraw from the study at any time. Following discontinuation, all participants’ individual study data will be deleted, and surveys and reminders will not be sent anymore.

#### Strategies to Improve Adherence to Interventions

To enhance adherence, the sign-up process for the digital solution was minimized. In addition, email reminders will be sent to patients. Moreover, the PRO feedback includes multiple elements (graphics, bolded text, and descriptions), as this was shown to improve patient understanding [27,32].

### Outcomes

The primary end point (research question 1) of the study is sufficient study participation to determine the representativeness of the results. As expected, study participation will vary depending on gender, age, and diagnosis, but this can be compensated for by weighting the study participants according to structural information on the target population.

Within our study, data on health care usage is available for both participants and the whole target population. Weighted measures of health care usage from participants will be compared with measures of health care usage of the complete target population and will be assumed to be formally representative if the weighted measures from participants fall within the 95% CI of the same measures based on data from the complete target population.

**Figure 2:**
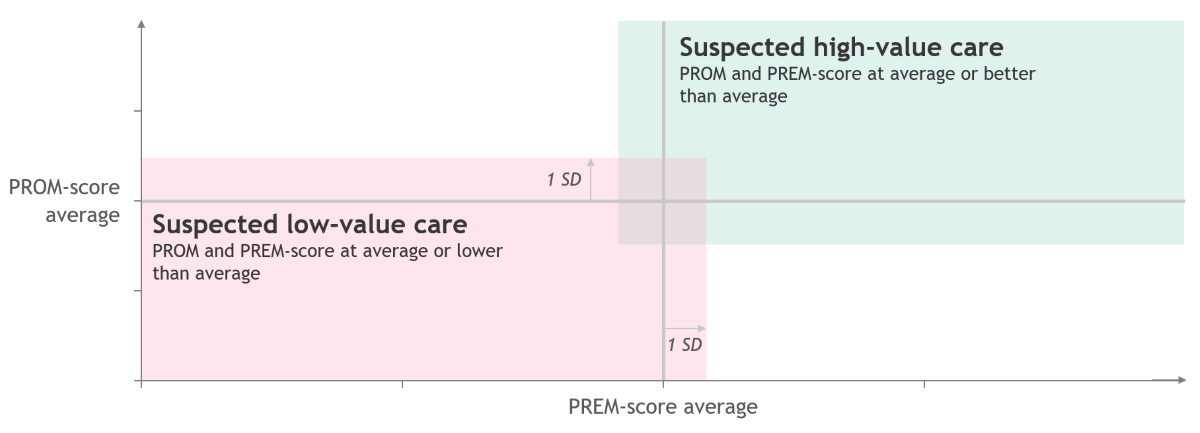
Assessment of value-based health care—outcome dimensions of suspected high-value and low-value care. PREM: patient-reported experience measure; PROM: patient-reported outcome measure.

Secondary end points are the end points to answer research questions 2 and 3 (Textbox 3; Figure 2).

Figure 2 represents how PROMs and PREMs can be combined into one value of care indicator, for example, if PREMs and PROMs are both average or above (up to 1 SD), care is suspected to be high-value, whereas if PROMs and PREMs are below 1 SD of the average, care is suspected to be low-value. Since for each disease group, a disease-specific PROM will be used next to a generic PROM, the assessment of low- and high-value care across diseases will be conducted by using the generic PROMs. Moreover, the disease-specific PROMs will be used to create low- and high-value care categorizations on a detailed level within a disease group. Adding cost data as a third dimension to the value of care framework (as described in the Statistical Analysis Plan, Multimedia Appendix 1) then enables a clear distinction between patients considered to have received low-value care and patients who received high-value care. A more detailed description of the outcomes can be found in the Statistical Analysis Plan (Multimedia Appendix 1).

**Research questions 2 and 3.**

**Research question 2:**

- Share of low-value care using the VBHC framework introduced in Figure 2 and specified in further detail in the Statistical Analysis Plan (Multimedia Appendix 1)
- Share of high-value care, specified in Figure 2
- Outcome and patient experience variation across subgroups
- Adverse events such as hospital admission due to a chronic disease profile

**Research question 3:**

- Comprehensibility and usability of patient-reported outcome (PRO) feedback
- Impact of PRO feedback on health care behavior

### Assignment of Interventions: Allocation

#### Sequence Generation

The sequence generation data are provided in Textbox 4.

The output is a data set containing matched pairs per indication. The first 25,000 matching pairs for each chronic condition will be selected. Diabetes type 1 and type 2 are considered jointly, with patients having diabetes type 1 selected first (approximately 6600 matched pairs are expected), followed by supplementing the remaining required matched pairs with patients having diabetes type 2.

**Sequence generation.**

- The BARMER health gender (male and female)
- 10-year age groups (first group: 18 to younger than 30 years, last group: 90 years and older)
  - Number of outpatient cases with indication diagnoses within the year 2021 (outpatient cases in less than 4 quarters, outpatient cases in all 4 quarters but less than or equal to 6 cases, outpatient cases in all 4 quarters with more than 6 cases)
- Matching Disease Management Program (DMP) and non-DMP patients by strata and a generated random numbering within the strata (1:1 matching).
- Consecutive selection of randomly sorted matched pairs if both matched individuals are insured all year long

#### Implementation

The random selection of patients will be performed by BARMER Health Insurance according to the selection process description provided by the independent evaluating aQua institute.

### Data Collection and Management: Plans for Assessment and Collection of Outcomes

There are 2 main data sources used in this study. First, primary data will be collected from the participants’ surveys. Second, secondary data from the cooperating health insurer, BARMER, will be used. The primary data collection is conducted via digital surveys, which include different questionnaire categories, namely PROMs, PREMs, comprehensibility of the PRO feedback, and questions on patients’ health behavior. Additionally, an anchor question using the Global Rating of Change scale [33] with 5 answer options is included to assess changes in health status.

**Figure 3:**
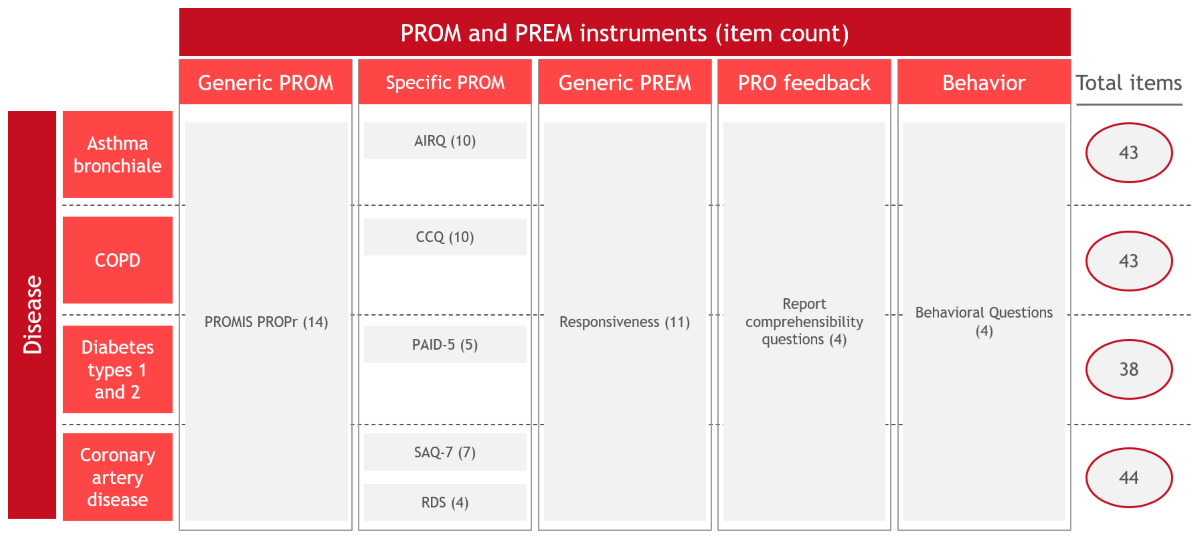
Survey items in the PROMchronic trial. AIRQ: Asthma Impairment and Risk Questionnaire; CCQ: Clinical COPD Questionnaire; COPD: chronic obstructive pulmonary disease; PAID-5: Problem Areas in Diabetes Scale–5; PREM: patient-reported experience measure; PRO: patient-reported outcome; PROM: patient-reported outcome measure; RDS: Rose Dyspnea Scale; SAQ-7: Seattle Angina Questionnaire-7.

An overview of all selected survey items, including the selected validated PROMs, can be found in Figure 3 and is described in more detail in the following paragraphs.

### PROMs

#### Overview

Various PROMs exist that can be classified into generic, treatment-specific, and disease-specific instruments. Generic PROMs assess health outcomes broadly and enable comparison across diseases, whereas treatment- or disease-specific PROMs are tailored to a specific treatment or disease and are more suitable for comparisons inside patient groups receiving the same treatment or a population with similar diseases [17]. The selection of the PROMs is in line with the selection criteria reported in the literature [34-37] as well as project-specific criteria. The criteria that were used for the selection of PROs are listed in Textbox 5.

**Criteria used for the selection of patient-reported outcomes (PROs).**

**Short**

- The questionnaire must be brief to not burden participants and increase response rates [38]. The overall item count across one survey period was restricted to 60 items and a maximum of 15 minutes to complete.

**Language**

- The patient-reported outcome measures (PROMs) must be available in German.

**Validation**

- The sets must be validated, at least in the German language, and ideally also for digital applications.

**Licensing fees**

- Licensing fee for surveys for academic use does not exceed US $5000 per set.

**Scoring**

- Scoring information must be available to enable PRO feedback based on overall scores.

**Health dimensions**

- The combined set of PROMs must cover all overarching health dimensions of physical, mental, and social health and reflect a mix of generic and disease-specific PROMs to enable informative intra- and inter-group comparisons.

**OECD comparison**

If possible, given the above-mentioned selection criteria, similar PROMs as the OECD (Organisation for Economic Co-operation and Development) PaRIS (Patient-Reported Indicator Surveys) initiative were selected to facilitate the comparability of results.

As a result, all participants will receive the PROMIS (patient-reported outcomes measurement information system) Preference Score (PROPr) [39] as well as disease-specific PROMs based on their chronic disease (Textbox 6).

**Disease-specific patient-reported outcome measures (PROMs).**

- Asthma: Asthma Impairment and Risk Questionnaire (AIRQ) [40]
- COPD: Clinical COPD Questionnaire (CCQ) [41]
- Diabetes: Problem Areas in Diabetes (PAID-5) [42]
- CAD: Seattle Angina Questionnaire-7 (SAQ-7) [43], and Rose Dyspnea Scale (RDS) [44]

#### PREMs

PREMs are used to collect information about the experiences of patients with health care services. PREMs ask patients about aspects of care such as communication, coordination, and access to care [19,45]. The responses can provide valuable insights into how health care services are perceived by patients and how they can be improved. There are a variety of different PREMs survey available, each with its own strengths and weaknesses and focus areas. Similar to the selection of the PROMs, the selection of PREMs followed criteria of time required to complete, validity, language, licensing fees, and HSPA relevance. Consequently, participants will receive the 11-item “responsiveness” component of the IPHA questionnaire with modified observation periods, as it is validated in chronic disease patients in Germany, is available in the German language, and is relatively short (11 items) [46].

#### Questions on PRO Feedback

The study examines a patient's perception and reaction to individual PRO feedback. First, patients will be asked if they have opened the pdf report. In addition, the comprehensibility and usefulness of the report will be investigated by asking 1 question each. A final question is asked about a possible change in mood after seeing the results in the pdf report. The set of questions can also be found in Textbox 7.

**List of questions on reactions to patient-reported outcome (PRO) feedback.**

**Review of report**

- Have you looked at your health report?

**Comprehensibility**

- Is the information in the health report understandable to you?

**Usefulness**

- Is the information in the health report helpful for you?

**Change in mood**

- How do you feel based on the information in your health report?

#### Health Behavior

Health behavior is significantly associated with health outcomes like mortality or the occurrence of chronic diseases [47]. Regarding chronic diseases, there are 5 essential health behaviors related to health outcomes, which include physical activity, diet, smoking, alcohol consumption, and sleep [48]. Participants’ perceptions of health behavior are assessed by 1 question on each of these 5 health behavior dimensions. Thereafter, their intention to change health behavior in the coming months and triggers for change are assessed. Additionally, participants receive a question regarding whether the results were discussed with their treating physicians.

#### Health Insurance Data

For those participants who have given consent, health insurance claims data will be linked to the participants’ primary data after the completion of the final survey period. Claims data will contain health care resource consumption (residence, comorbidities, inpatient hospital stays, outpatient consultations, complications, rehabilitation, drugs, physiotherapy, medical remedies and aids, and care services) and additional data points further specified in the Statistical Analysis Plan (Multimedia Appendix 1).

#### Plans to Promote Participant Retention and Complete Follow-Up

Complete survey data is important to calculate PROM scores [49]. Participants can access digital dashboards showing open tasks (questionnaires) and the remaining time to complete them. To increase data completeness, the participants will be reminded by email to fill in their follow-up questionnaires 1, 3, and 10 days after the initial dispatch of surveys.

#### Data Management

The collection, storage, and processing of personal data in this project are carried out in accordance with the General Data Protection Regulation in Germany, the specific data protection provisions of the Social Code, and all other national data protection regulations. During the study, all electronically recorded primary data, as well as participation and consent forms, will be stored on the server of Oncare GmbH and deleted after the end of the evaluation period. The Oncare team will manage patient information through the myoncare app and study website, always respecting data security and confidentiality. All reading and processing processes are logged in the database. All data will be collected and transferred completely pseudonymized.

Pseudonyms are created by the health insurer BARMER following the universally unique ID standard and are only re-identifiable by BARMER health insurance. Consequently, the pseudonym is added to the health insurance data to match primary patient data to insurance claims data. All identification information will be erased prior to data transfer to the research institutes Technical University Berlin and aQua Institute. Pseudonymized data will be kept for the period of data analysis of 2 years by the Technical University Berlin and aQua Institute and stored for an additional 10 years at study centers to ensure further evaluation of the study's outcome. This follows the recommendations for good practice in secondary data analysis [50].

#### Confidentiality

A unique study pseudonym is assigned to each participant by the health insurer. The pseudonym list with patient names will only be accessible to the health insurer, and they will not receive any primary data. During the primary data collection, no data that would allow re-identification will be collected. Minimum contact data is collected to ensure follow-up surveys can be completed and reminders can be sent to the participants. Linkage of health insurance data will be conducted via study pseudonyms. The project adheres to all data protection laws.

### Statistical Methods

#### Statistical Methods for Primary and Secondary Outcomes

The statistical analyses are reported in the separate Statistical Analysis Plan (Multimedia Appendix 1) but generally include descriptive statistics, parametric and nonparametric methods, as well as time series analyses for the primary and secondary end points.

#### Methods for Additional Analyses (eg, Subgroup Analyses)

**Figure 4:**
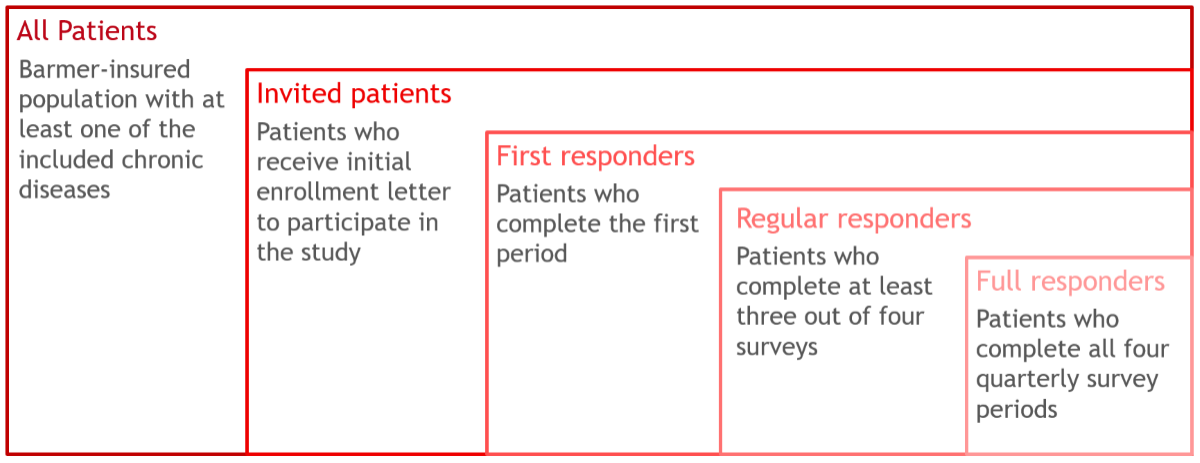
Definition of study population and patient groups based on response status for statistical analysis.

Participants with the respective chronic diseases are grouped into specific subgroups based on the research questions. For all participant groups, additional control data from overall insured patients will be accessible on an aggregated level. Therefore, the study population is segmented into different subgroups (Figure 4 and Textbox 8).

**Subgroups of study population.**

- All patients: a full BARMER-insured population with at least one of the chronic diseases included in the study, with data available only at an aggregated level
- Invited patients (from “All patients”): Patients who receive the initial invitation letter
- First responders (from “Invited patients”): Patients who complete the first survey period, including consent to use their health insurance data
- Regular responders (from “First responders”): Patients who complete at least 3 out of 4 survey periods
- Full responders (from “Regular responders”): Patients who complete all 4 quarterly survey periods

#### Methods in Analysis to Handle Protocol Nonadherence and Any Statistical Methods to Handle Missing Data

The study is set up as a prospective observational cohort study, inviting patients to complete different surveys over a 1-year time frame. Nonadherence, defined as nonparticipation or dropout in this study, is one of the main research questions and will not be handled specifically for research question 1. Complete PROM data are important to calculate scores. In cases of missing data, we will adhere to PROM-specific guidelines to handle missing data (eg, imputation or calculating scores based on remaining values).

## Results

### Composition of the Coordinating Center and Trial Steering Committee

The study is monitored by the German research center “Deutsches Zentrum für Luft- und Raumfahrt” (German Aerospace Center). Quarterly status reports are provided by the project team. Status reports include an overview of the achievement levels of the milestones defined prior to the start of the study.

### Composition of the Data Monitoring Committee, Its Role, and Reporting Structure

The management of the study is overseen by a project team from Technical University Berlin. The project team is composed of researchers responsible for the study's design, representatives from the participating BARMER health insurance, the technical service provider Oncare, and the evaluating aQua Institute. Regular updates on the study's status are provided to the sponsor.

### Adverse Event Reporting and Harms

Adverse events are not expected as the medical treatment of patients with chronic care is not affected. The effects of the PRO feedback intervention are one of the primary end points, and the results will be monitored and published.

### Plans for Communicating Important Protocol Amendments to Relevant Parties (eg, Trial Participants and Ethical Committees)

Any substantial amendments to the protocol will be submitted to the ethics committee (see below) and all relevant regulatory institutions. Additionally, any amendments to the study design, timeline, or budget need to be communicated to the study sponsor, and approval by the coordination center must be obtained.

### Dissemination Plans

The results of this study will be disseminated via publications in peer-reviewed journals and presentations at relevant conferences. Moreover, the funding institution (Innovation Fund of the Federal Joint Committee) will receive an evaluation report that includes the findings of the study as well as interim reports on the study’s milestones. All results will be aggregated, with no opportunity to reconnect on an individual patient level.

## Discussion

### Principal Findings

The study aims to fill the gap in the literature on large-scale usage of ePROMs and ePREMs in patients with chronic diseases. First, the study protocol details how the representativity of the responses to ePROMs and ePREMs will be assessed over time in different subgroups. This foundational groundwork will help with future targeted efforts and the mitigation of representativity issues. Second, the study protocol identifies how PROMs and PREMs will be used to uncover potential low-value care for patients with chronic diseases. The results can thus inform future investigations on activities for low-value care reduction. Third, it introduces how individual PRO feedback can be shared with patients with chronic diseases and provides insights into the usability and usefulness of PRO feedback. The results of the evaluation can thereby aid the understanding of this specific PRO feedback mechanism.

ePROM collection will increasingly be the standard for capturing patients’ perspectives on treatment outcomes as well as their own health status. It was previously shown that the administrative burden for patients and health care providers can be significantly reduced through the collection of ePROMs compared with phone- or paper-based collection, while response rates and the completeness of data collection remain high [51]. The results of this study will fill the research gap in terms of the representativity of the patient population through ePROM and ePREM collection, for which no previous evidence exists. While this study uses ePROM to measure care quality variation, most of that research was focused on oncological studies [52]. This study aims to fill the research gap on measuring care quality through ePROM and ePREM usage in patients with chronic diseases. Other studies have shown, that working with PROMs can improve care by significantly reducing low-value behavior [53,54]. Additionally, the use of ePROMs and ePREMs can enable (almost) real-time, individual PRO feedback [55-57]. Providing PRO feedback is one way to enhance the use of PROMs in clinical practice and shared decision-making [58]. Previous studies indicate that patients who reviewed shared information on their PRO outcomes are more engaged and actively participate in their health care, but they have not investigated direct-to-patient feedback in patients with chronic diseases [59,60].

There are some limitations to the study that need to be considered. One limitation is the potential for nonresponse bias, as patients who choose to participate in the study may differ from those who do not. However, given the access route via large-scale, randomized health insurance paper-based outreach, we hope some participants who would not take part in studies in a clinical study will be accessed. Moreover, it is one of the study’s aims to detect the representativeness of the responders. Given that the letter and questionnaire will be in German, we anticipate that non-German speakers will be excluded from the study, which unfortunately could not be addressed via the digital solution, the adjustment to letters, and the low availability of validated PROMs in other languages often spoken besides German in Germany (eg, Russian or Turkish). Moreover, respondents to the study might do systematically better in terms of their PROMs and PREMs results, which cannot be assessed if there are no other systematic differences between the responder and nonresponder population [61]. This could also be the case if the monitoring itself improves health outcomes, as suggested in the current literature [62,63].

The broader implications of this study are insights generated for (1) ePROM and ePREM usage among patients with chronic diseases globally and specifically in Germany, as well as (2) cross-country learnings. (1) The expected benefits of PREM and PREM usage among patients with chronic diseases were previously stated by experts, while a lack of evidence in the treatment of chronic diseases exists [22]. This study will examine if and to what extent digitally collected PROMs and PREMs as well as automatically generated PRO feedback could strengthen patient empowerment, informed shared decision-making, and behavior changes in patients with chronic diseases. (2) Given that the OECD PaRIS study will assess PROMs and PREMs across many countries besides Germany, the results of this study can enable Germany to be part of this research community and to benchmark its health care system against those of other OECD countries. Long-term benchmarking results across countries and within Germany can have implications for health care spending based on patients’ needs and care reorganization while raising awareness of care value.

### Trial Status

This protocol is version 1, dated October 5, 2023. Patient recruitment will begin with BARMER's letter dispatch around October 11, 2023. The collection of survey data will be finished by September 30, 2024. The study is expected to run until June 30, 2025.

## Appendix

Multimedia Appendix 1 Statistical Analysis Plan.

Multimedia Appendix 2 Invitation letter for participation in the study.

Multimedia Appendix 3 Study Information and Consent Form.

Multimedia Appendix 4 Patient-reported outcome feedback.

## Abbreviations

CAD: coronary artery disease
COPD: chronic obstructive pulmonary disease
DMP: Disease Management Program
ePREM: electronic patient-reported experience measure
ePROM: electronic patient-reported outcome measure
HSPA: health system performance assessment
OECD: Organisation for Economic Co-operation and Development
PaRIS: Patient-Reported Indicator Surveys
PREM: patient-reported experience measures
PRO: patient-reported outcome
PROM: patient-reported outcome measures
PROPr: PROMIS (patient-reported outcomes measurement information system) Preference Score

## Data Availability

The data sets generated and analyzed during this study are available from the corresponding author on reasonable request.
