# Supplementary material for: Evaluating the Population-Based Usage and Benefit of Digitally Collected Patient-Reported Outcomes and Experiences in Patients With Chronic Diseases: The PROMchronic Study Protocol
Source: JMIR Res Protoc. 2024 Aug 5;13:e56487. doi: 10.2196/56487 (PMC11333866; doi:10.2196/56487)
Supplement: Multimedia Appendix 1 [file resprot_v13i1e56487_app1.docx]

**Protocol Version 1**

**Evaluating the population-based utilization and benefit of digitally collected Patient-reported outcomes and experiences in patients with chronic diseases: The PROMchronic study**

Document Version History

| Version Date | Version | Author | Signature | Change Description | Reason/Comment |
| --- | --- | --- | --- | --- | --- |
| 05/10/2023 | 1 | JN |  | Initial release. | Not applicable. |
|  |  |  |  |  |  |
|  |  |  |  |  |  |

**TABLE OF CONTENTS**

[LIST OF ABBREVIATIONS 3](#__RefHeading___Toc289353016)

[1. Study Objectives 4](#__RefHeading___Toc289353017)

[1.1. PRIMARY OBJECTIVE 4](#__RefHeading___Toc289353018)

[1.2. SECONDARY OBJECTIVES 4](#__RefHeading___Toc289353019)

[2. Background/Introduction 4](#__RefHeading___Toc289353020)

[2.1. STUDY DESIGN 4](#__RefHeading___Toc289353021)

[2.2. TREATMENT GROUPS 4](#__RefHeading___Toc289353022)

[2.3. STUDY POPULATION 4](#__RefHeading___Toc289353023)

[2.4. INTERVENTION 4](#__RefHeading___Toc289353024)

[2.5. SAMPLE SIZE 4](#__RefHeading___Toc289353025)

[2.6. STUDY PROCEDURE 4](#__RefHeading___Toc289353026)

[3. Populations of Analysis 5](#__RefHeading___Toc289353027)

[4. Outcome Variables 5](#__RefHeading___Toc289353028)

[4.1. PRIMARY OUTCOME 5](#__RefHeading___Toc289353029)

[4.2. SECONDARY PARAMETERS OUTCOMES 5](#__RefHeading___Toc289353030)

[4.3. OTHER PARAMETERS 5](#__RefHeading___Toc289353031)

[5. Statistical Methodology 5](#__RefHeading___Toc289353032)

[5.1. GENERAL METHODOLOGY 5](#__RefHeading___Toc289353033)

[5.2. PRIMARY DATA ANALYSES 6](#__RefHeading___Toc289353034)

[5.3. SECONDARY DATA ANALYSES 6](#__RefHeading___Toc289353035)

LIST OF ABBREVIATIONS

AIRQ Asthma Impairment and Risk Questionnaire

BMI Body-Mass-Index

CAD Coronary artery disease

CCQ Clinical COPD Questionnaire

COPD Chronic obstructive pulmonary disease

DMP Disease management program

ePREM Digital patient-reported experience measure

ePROM Digital patient-reported outcome measure

HSPA Health-system performance assessment

ICD International statistical classification of diseases and related health problems

OPS Operationen- und Prozedurenschlüssel [German procedure classification]

PAID Problem Areas in Diabetes

PROM Patient-reported outcome measure

PREM Patient-reported experience measure

SAQ Seattle Angina Questionnaire

RDS Rose Dyspnea Scale

1. Study Objectives
   1. PRIMARY OBJECTIVE

Our study aims to evaluate the usability and potential benefits of the structured use of patient-reported outcome measures (PROMs) and patient-reported experience measures (PREMs) to improve care for patients with chronic diseases in Germany.

**Main research question:**

Can digital PROMs (ePROMs) and digital PREMs (ePREMs) be used in patients with chronic diseases for quality measurement at the health system level (e.g., for health-system performance assessment (HSPA))?

- How representative are response rates in patients with chronic diseases via digital surveys?
- How do response rates and willingness to respond multiple times vary over time by age, gender, indication, city/state, health system use (frequent versus infrequent users), web- or app-based surveys, and Disease Management Program (DMP) participation?
  1. SECONDARY OBJECTIVES

1. To what extent can value of care variation in care be identified from ePROMs and ePREMs surveys?

- What is the share of suspected low-value care and suspected high-value care in Germany, per indication and in subgroups?
- How do ePROM and ePREM results differ according to age, gender, indication, city/state, healthcare system utilization (changes in frequency of outpatient practitioner attendance, prescriptions, hospitalizations, etc.), and DMP participation?
- Can ePROMs and ePREMs function as an early warning signal for deteriorating chronic conditions or adverse events such as hospital admission?

1. What are the benefits or drawbacks of PRO feedback (outcome reports) sent to patients?

- Are the PRO value reports understandable to patients?
- Can PRO feedback function as a positive nudge (lead to positive behavioral changes e.g., healthier or more active lifestyle, more active participation in medical treatment, actively approaching treating physicians in the office setting)?
- Are there any negative emotional reactions when receiving PRO feedback that shows values worse than those of a comparable group?

1. Background/Introduction
   1. STUDY DESIGN

The study is an observational prospective cohort study with patients of four chronic disease areas with existing DMPs (asthma, chronic obstructive pulmonary disease (COPD), diabetes (type 1 and type 2) and coronary artery disease) with two sub-cohorts (DMP and non-DMP) for each disease.

- Type of control: none / group matched BARMER insurees (matching criteria: disease area, gender, age group)
- Level and method of blinding: none
- Method of treatment assignment: stratified randomized selection of study population
- Number of subjects: 200,000 patients invited (50,000 per chronic indication and about 25,000 in each sub-cohort)
- Duration of study: one year (four quarterly survey periods)
  1. TREATMENT GROUPS

There are four disease cohorts with two sub-cohorts, each. The first sub-cohort consists of subjects participating in a DMP. The subjects of the second sub-cohort are not participating in any DMP but have a documented ICD-10-GM diagnosis of one of the selected diseases.

The ICDs of the second sub-cohorts are defined as:

- Diabetes mellitus type 1: E10, diabetes mellitus type 2: E11
- COPD: J44
- Asthmatic disease: J45
- Coronary artery disease: I25
  1. STUDY POPULATION

Inclusion criteria:

- Patient is at least 18 years old
- At least two outpatient consultations documenting a confirmed chronic disease diagnosis in 2021
- For type 1 diabetes at least one insulin prescription must be present in 2021

Exclusion criteria:

- Participation in more than one DMP
  1. INTERVENTION

In addition to filling out the survey questions at four points in time (intervention 1), all participants will receive PRO feedback as a potential nudge (intervention 2). The PRO feedback will be a pdf report sent via email and available in their study participant profile, which graphically (line charts) shows the patients’ individual generic and disease-specific health status in comparison to the individual peer group. In addition to the visual feedback, the results will shortly be explained on the same page. The description will cover whether patient-individual PRO scores are better or worse compared to the peer group, and for longitudinal values, whether patient-related changes improved or worsened. Additional details on the scores, sub-scores, and calculations are shared with the patients on the following pages of the report. Peer groups are classified according to their disease, gender, and age group. The peer group is created based on individuals who have replied to the previous survey and will only be displayed if the group consists of at least four individuals. This threshold is set by the German General Data Protection Regulation. The report refers to the patients’ physicians as the main contact to discuss the results or in case of questions.

- 1. SAMPLE SIZE

The sample is drawn stratified randomly from insured patients of one large German statutory health insurance with about 9 million insured persons (of about 73 million statutorily insured persons in total in Germany) according to their diagnosis and/or disease management program status. A one-to-one matching of participants of one DMP with non-participants of a DMP but with the same proven diagnosis according to the ICD-10-GM system is made. Further stratification criteria are age group, gender, and health system utilization.

An initial invitation letter is sent to 200,000 insured individuals, 50,000 of each of the selected chronic conditions (about 25,000 per sub-cohort). Diabetes type 1 and type 2 are considered jointly as there is no differentiation in the selected PROMs. The patients of all chronic diseases are allocated to groups based on their participation in DMP (DMP or non-DMP group). Prior experiences suggest that around 30% of patients react to invitations to participate in research by their insurer [29-31]. Two-thirds of initial participants are expected to allow follow-up contacts and around 40% of these patients will continue participating in all the follow-up surveys after receiving reminders for each task. Therefore, after accounting for non-participants and participants with no complete survey data we expect a complete data set for 16,000 participants. However, the first research question will investigate response rates as there is no evidence on this population yet.

- 1. STUDY PROCEDURE

Eligible participants will be participating in the study for a maximum of four quarters, starting with the first access of the digital questionnaires followed by quarterly digital surveys split into several tasks. To start the survey, participants register with their study pseudonyms and share personal information, e.g., basic demographics and identification data. Each survey period contains four tasks to be completed by participants defined as sets of questions that each will take five to ten minutes to complete. The participants are invited to answer PROM and PREM surveys.
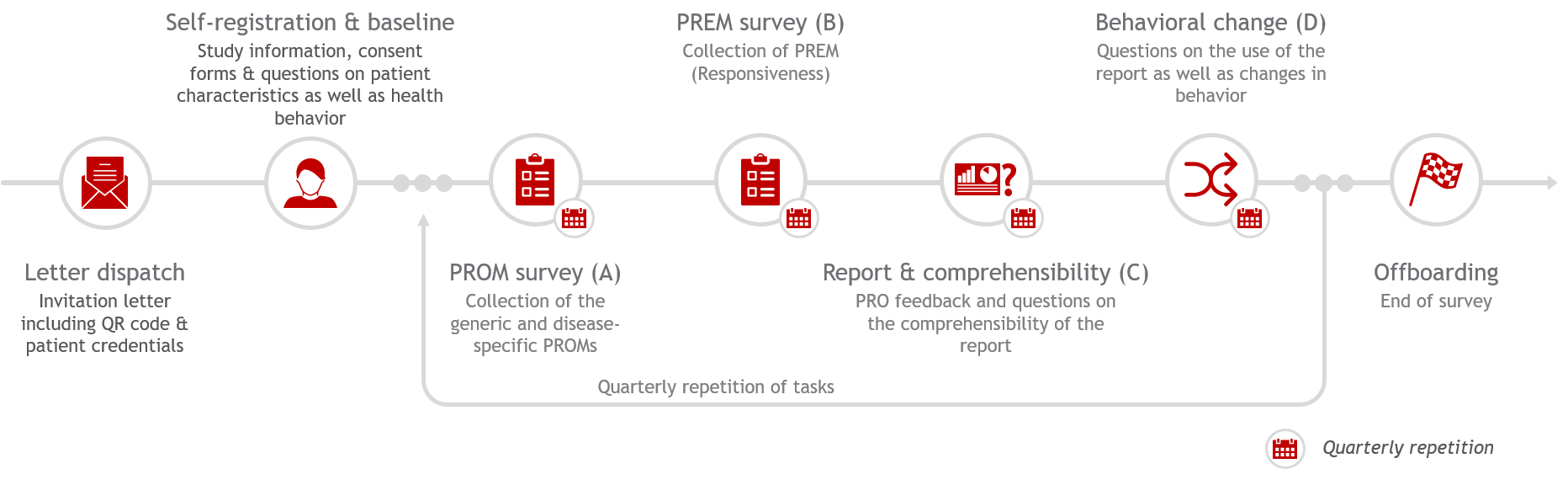


*Figure 1: Study design: Participants’ timeline and tasks*

As shown in Figure 1, the first point of contact will be a letter from the insurance to insured individuals with chronic diseases, who can then sign into the digital study via a QR code or link. For each of the different disease areas, a distinct care path is opened, with a sign-in process followed by selected PROMs and PREMs. The study covers a timeframe of one year with four overarching time intervals with dedicated questions. These are split into smaller tasks to facilitate the answering process for participants. The time intervals are split into smaller tasks to facilitate the answering process for participants. A few weeks after responding to the first set of questions (A & B), patients will receive a report with their individual PROM scores compared to a peer group, the PRO feedback (C). From the second interval onwards, patients will also receive the PRO feedback in a longitudinal visualization. Lastly, following the PRO feedback participants will be surveyed regarding their health behavior (D).

Figure 2 presents the questionnaires that will be used in the study per chronic disease group. PROM surveys include generic as well as disease-specific PROMs. The participants will receive individual reports on their patient-specific and peer group outcomes (PRO feedback) and will be asked about the comprehensibility of the report. Following the report, the participants receive a set of questions regarding the report’s comprehensibility and their health-related behavior.


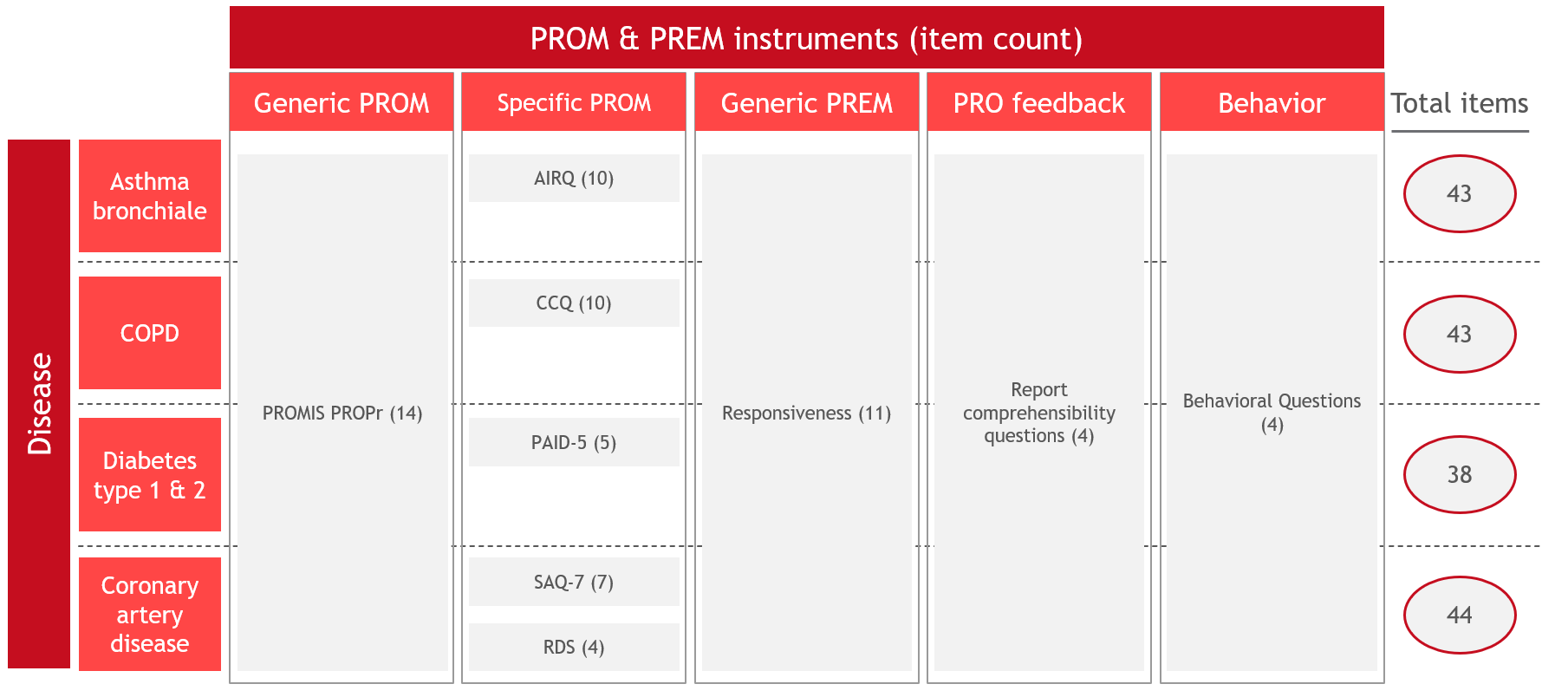


*Figure 2: Survey items in the PROMchronic trial*

The primary data collected in this study is hence:

**Patient reported indicators:**

- Generic PROM index score: PROMIS PROPr is a preference-based score which combines seven PROMIS domains of health status into one score (cognitive function, depression, fatigue, pain, physical function, sleep, social roles)
- Disease-specific PROM index scores for each chronic disease

Asthma: Asthma Impairment and Risk Questionnaire (AIRQ) score

COPD: Clinical COPD Questionnaire (CCQ) score

Diabetes: Problem Areas in Diabetes (PAID-5) score

Coronary artery disease: Seattle Angina Questionnaire (SAQ-7) score and Rose Dyspnea Scale (RDS) score

- PREM index score: Responsiveness of the health care system
- Looked at PRO feedback
- Comprehensibility of PRO feedback
- Helpfulness of PRO feedback
- Emotional reaction to PRO feedback
- Discussion of PRO feedback with physician(s)
- Health behavior in five dimensions:

Physical activity

Nutrition

Tobacco consumption

Alcohol consumption

Sleep duration

- Plans to change health behavior in the future
- Trigger for health behavioral change
- Overall health improvement
- Disease-specific health improvement

**Patient reported demographic information:**

- Age: continuous variable in years measured as dates (DD/MM/YYYY)
- Gender: categorical variable (male, female, diverse, I do not want to say)
- Height: continuous variable measured in cm
- Weight: continuous variable measured in cm
- Education: categorical variable (without general school certificate, primary or secondary school, university entrance qualification, bachelor’s degree, master’s degree, doctorate, other)
- Occupation: categorical variable (Self-employed, employed (full-time, part-time, mini job), student, apprentice, unemployed (jobseeker, homemaker, unable to work due to illness, retired), other)
- Household income: categorical variable (<749€, 750 – 999€, 1,000 – 1,499€, 1,500 – 1,999€, 2,000 – 2,499€, 2,500 – 3,499€, 3,500 – 4,499€, 4,500 – 5,499€, >5,500€, I do not want to say, I do not know)
- Household size: categorical variable (1, 2, 3, 4, 5, 6 or more)
- Household size under 14 years: categorical variable (0, 1, 2, 3, 4, 5 or more)

**The secondary data, retrieved from the participating health insurance BARMER includes the following data points:**

- Personal data: age, sex, death date (if applicable), insurance times, region of residence
- Disease management program participation: program type, participation times
- Outpatient care data: date of attendance, invoiced fee schedule items, practitioner’s specialty, diagnoses (ICD-10-GM), procedures (OPS)
- Prescriptions of medications: drug identifier, no of prescriptions, date of prescription, prescribing practitioners’ specialty, anatomic-therapeutic-code (ATC), dosage (defined daily dosage - DDD), costs
- Allied health professions (podiatric, dietary, speech and language, ergo- and physiotherapy): date of prescription, indication, diagnosis (ICD-10-GM), prescription type, remedy type, first/last date of remedy application, number of applications, prescribing practitioners’ specialty, costs
- Assistive technology: date of prescription, unique identifier of assistive technology, first/last date of application, number of applications, prescribing practitioners’ specialty, costs
- Inpatient hospital stays: clinic identifier, date of admission/discharge, admission/discharge mode, discharging departments’ specialty, diagnoses (ICD-10-GM), procedures (OPS)
- Hospital care: clinic identifier, date of entry/discharge, diagnoses (ICD-10-GM), procedures (OPS), fee schedule items, costs
- Work incapacitation: first/last date of work incapacitation, diagnosis (ICD-10-GM), days of wage-replacement-benefits, costs of wage-replacement-benefits
- Transportation: prescribing practitioner with specialty, prescription date, first/last day and number of service(s), fee schedule items, costs
- Household assistance: prescribing practitioner, prescription date, first/last day and number of service(s), fee schedule items, costs
- Short-term nursing care: prescribing practitioner, prescription date, first/last day and number of service(s), fee schedule items, costs
- Rehabilitation: prescribing practitioner, first/last day of rehabilitation, type of rehabilitation (inpatient/outpatient, mobile, combinations), funding agency, diagnoses (ICD-10-GM), costs
- Long-term care benefits: level of care, first/last date of the current level of care, care type, first/last date of care benefits, costs

1. Populations of Analysis

All patients who were selected in the identification of study population and participated in the study except for patients who are excluded from the study due to:

- Consent withdrawal

All the analyses will be performed on this population.

1. Outcome Variables
   1. PRIMARY OUTCOME

The primary endpoint of the study is the sufficient study participation to determine the representativeness of results for first, regular and full responders (compare figure 3).


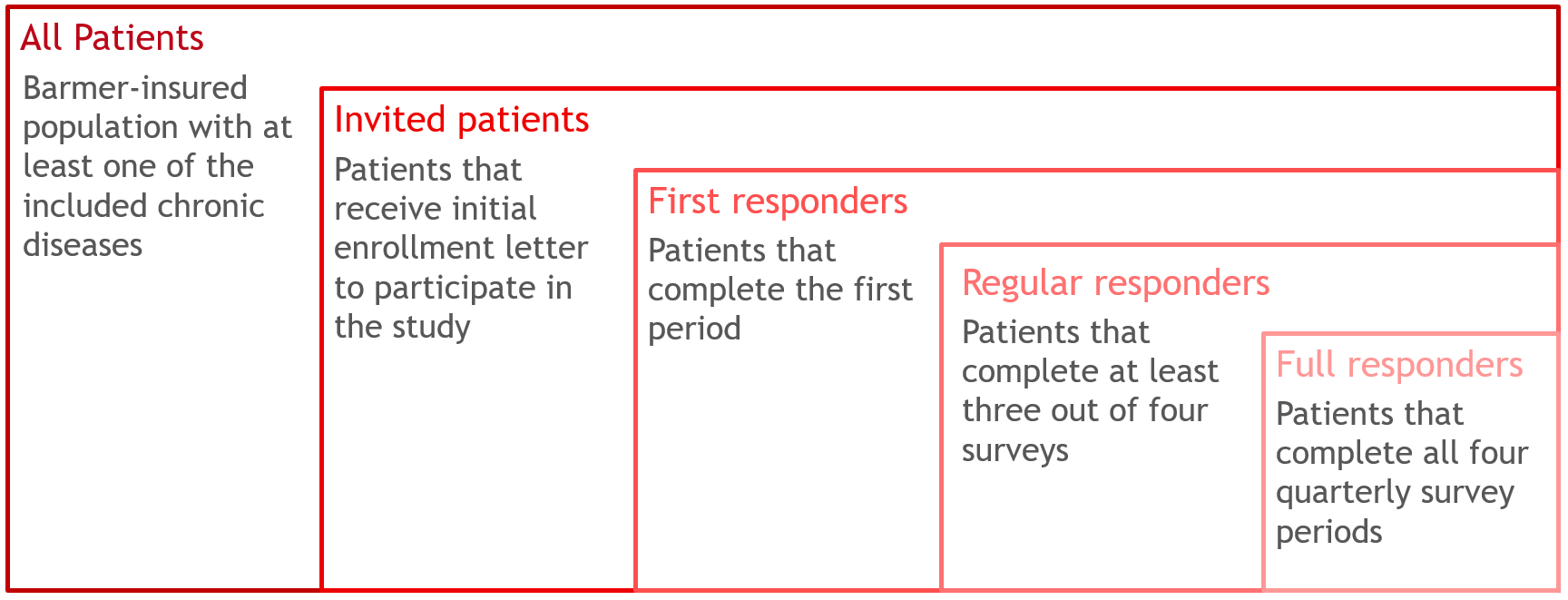


*Figure 3: Overview of patient and participant segmentation*

As expected, study participation will vary depending on gender, age, and diagnosis, but this can be compensated for by weighting the study participants according to structural information on the target population. Within our study, data on healthcare utilization is available for participants and the whole BARMER population. Weighted measures of health care utilization from participants will be compared with measures of health care utilization of the complete target and will be assumed to be formally representative if the weighted measures from participants fall within the 95% confidence interval of the same measures based on data of the complete target population.

- 1. SECONDARY PARAMETERS OUTCOMES

**For the primary research question:**

As secondary outcome for the primary research question, (1) the response rates overall and per patient subgroups based on disease area, patient characteristics (gender, age, disease), DMP participation, and others as well as (2) the representativity of responding participants over time. For these analyses, the study population is segmented in:

- All patients: Full BARMER-insured population with at least one of the chronic diseases included in the study
- Invited patients (from “All patients”): Patients that receive the initial invitation letter
- First responders (from “Invited patients”): Patients that complete the first survey period
- Regular responders (from “First responders”): Patients that complete at least three out of four survey periods
- Full responders (from “Regular responders”): Patients that complete all four quarterly survey periods

Variables that will be analyzed in all segments:

- Age
- Gender
- Type of chronic disease
- DMP participation

For all responder groups, additional variables from survey data will be analyzed:

- Body-Mass-Index (BMI), calculated by height & weight
- Education
- Profession
- Household size
- Household income

**For the second research question:**

- Share of low-value care (see Statistical Analyses section)
- Share of high-value care (see Statistical Analyses section)
- Outcome and patient experience variation across subgroups
- Adverse events such as hospital admission due to chronic disease profile

**For the third research question:**

- Comprehensibility of PRO feedback
- Helpfulness of PRO feedback
- Impact of PRO feedback on healthcare behavior overall and per subdimension
  1. OTHER PARAMETERS

**Demography and Baseline**

See Secondary Parameter Outcomes in 4.2.

**Safety**

Not applicable

**Laboratory**

Not applicable

1. Statistical Methodology
   1. GENERAL METHODOLOGY

Quantitative variables will be described as mean (respectively median), standard deviation (inter-quartile range), number, and rate of missing data, and compared between arms with a t-test, or if not appropriate with a Wilcoxon rank-sum test. Qualitative variables will be reported as absolute and relative frequencies, number, and rate of missing variables, and compared between sub-cohorts with the Chi-square test or the Fisher’s exact test. Variables will be compared between both sub-cohorts (DMP and non-DMP participants). P-value <0,05 with a bilateral hypothesis will be considered significant. All the analyses will be carried out using R, SAS, and other statistical software for Windows.

**Handling of missing data**

For the analysis of the primary research question missing data is reported as NA and no imputation will be performed. The number of missing data points will be reported for each variable and survey period. A summary of missing variables and effects due to incompleteness is reported in the related publications.

For the analysis of the second and third research questions, applicable outcomes will be imputed using missing Forest imputation.

**Sensitivity analysis**

Sensitivity analyses will be performed for the outcomes (see primary and secondary outcomes in 4.1 and 4.2).

**Subgroup analysis**

Subgroup analyses will be conducted by all patient characteristic variables under 4.2. Height & weight will be combined to BMI according to WHO standards. Additionally, subgroup analyses will be performed by DMP membership and healthcare utilization. Subgroup analyses cover all study participants as well as analyses of each chronic disease population.

**Classification of protocol violation**

Not applicable

- 1. PRIMARY DATA ANALYSES

**Primary Outcomes**

*Sufficient response rates for representativity of subgroups*

As expected, study participation will vary depending on gender, age, diagnosis, and healthcare utilization, but this can be compensated for by weighting the study participants according to structural information on the target population.

Within our study, data on the above-mentioned patient characteristics is available for both participants and the whole target population. Weighted measures from participants will be compared with measures of the complete target and will be assumed to be formally representative if the weighted measures from participants fall within the 95% confidence interval of the same measures based on data of the complete target population.

The general methodology (see 5.1) will be applied for the analysis of the primary outcomes.

**Secondary Outcomes**

Value of care:

Value of care delivery is assessed according to the Porter value-based healthcare framework:

Patient-Value = Outcome/Costs

In the analysis, we will firstly focus on the outcome side of the value equation and assess the outcome side based on PROMs and PREMs. The categorization into suspected high-value and suspected low-value care is hence based on PROMs and PREMs. Outcomes higher than average in at least two survey periods are classified as suspected high-value care whereas outcomes lower than average in at least two survey periods are classified as suspected low-value care. Outcomes not included in one of the categories will be separated and excluded from analysis (see Figure 4).


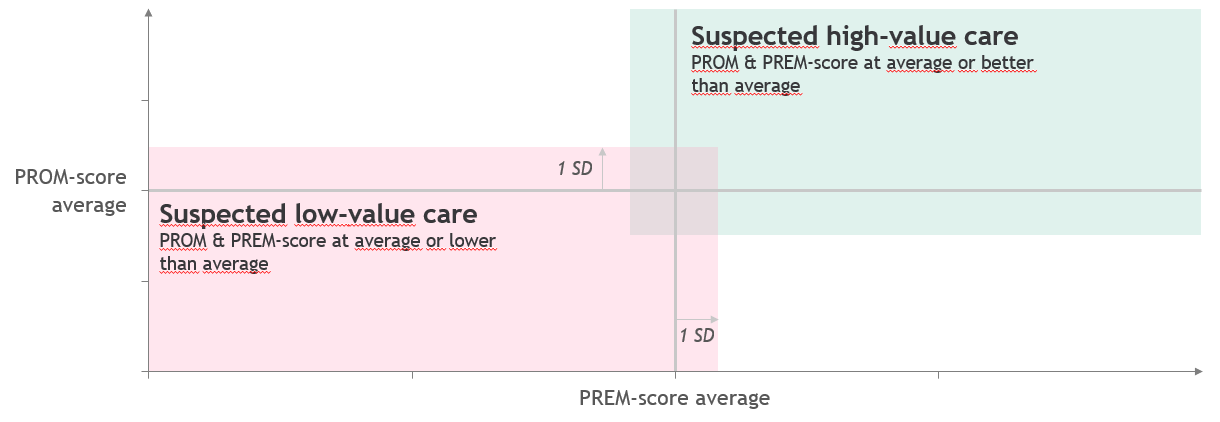


*Figure 4: Assessment of Value-Based Healthcare - Outcome dimension*

|  | **PROM – outcome (health)** | **PREM – outcome (responsiveness)** |
| --- | --- | --- |
| **Suspected low-value care** | Maximum one standard deviations above average in at least two survey periods | Maximum one standard deviations above average in at least two survey periods |
| **Suspected high-value care** | Maximum one standard deviations below average in at least two survey periods | Maximum one standard deviations below average in at least two survey periods |
| **Measure-ment** | Separately for health-related quality of life (PROPr index score) & disease-specific health (index score of the disease-specific PROM):   - COPD-related health status - Asthma-related health status - Coronary artery disease-related health status - Diabetes-related health status (for both types) | German responsiveness questionnaire index score |

*Table 1: Assessment of Value-Based Healthcare - Outcome dimensions and measurement*

To cover the cost-side of the value equation as well, we add the health care system utilization. For each of the chronic diseases average treatment costs will be calculated. Costs will cover all healthcare related costs (see 2.6 secondary data). Depending on the deviation from the average participants are classified in the following categories:

- Low costs: More than one standard deviation below average in at least two survey periods
- High costs: More than one standard deviation above average in at least two survey periods
- Inconspicuous costs: Else

Based on the combination of PROMs, PREMs and health care system utilization measured as costs, a value-based healthcare cube is introduced. PROM and PREM scores function as outcome dimensions and healthcare system utilization as cost dimension. Costs are hence added as new axis inside the framework (see Figure 5).


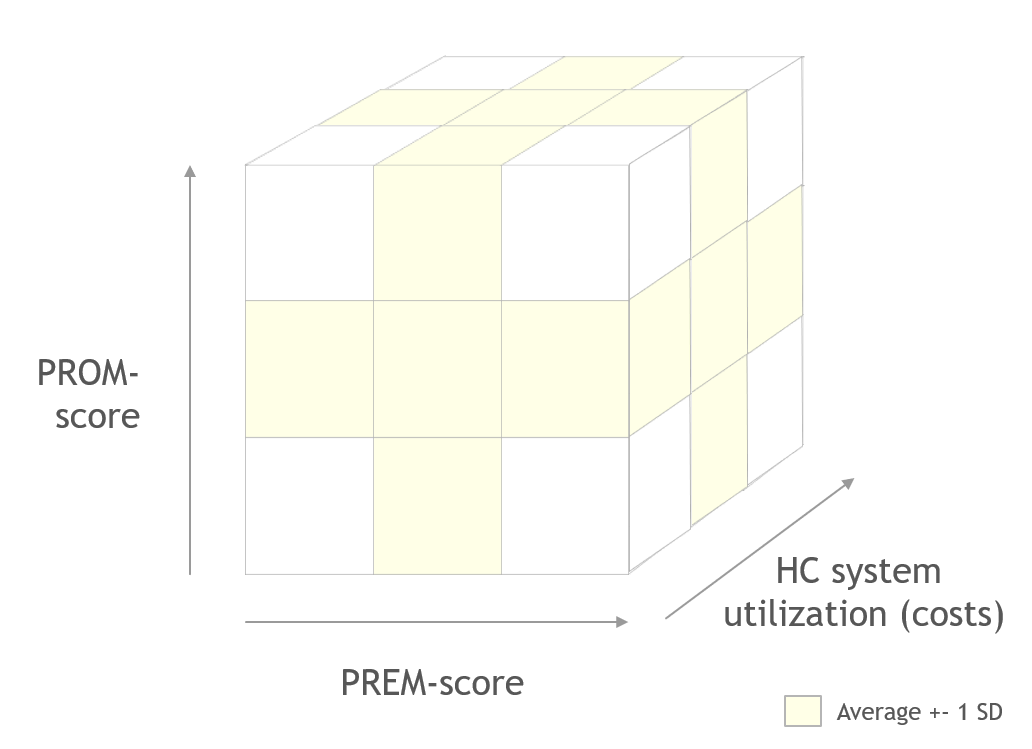


*Figure 5: Assessment of Value-Based Healthcare - Framework*

The newly introduced value-based healthcare cube allows the measurement of high-value and low-value care. Along the previously defined areas of suspected high- and low-value care (see Figure 4 and 5), the cube can be subdivided into 27 segments (see Figure 6). High-value and low-value care will be investigated based on the following criteria:

- High-value indication in both dimensions (high patient-relevant indicators (PROMs/PREMs) **at** low costs) (Figure 6 – dark green area)
- High-value indication in at least one dimension (high patient-relevant indicators (PROMs/PREMs) **or** low costs) (Figure 6 – light green area)
- Low-value indication in at least one dimension (low patient-relevant indicators (PROMs/PREMs) **or** high costs) (Figure 6 – light red area)
- Low-value indication in both dimensions (low patient-relevant indicators (PROMs/PREMs) **at** high costs) (Figure 6 – dark red area)

The value of care dimensions will be summarized into high- versus low-value care as the broad definition and into high two-sided, high one-sided, low one-sided and low two-sided value care as narrow definition. For both categorizations the frequency as well as the variation per subgroups (e.g. region, DMP etc.) will be analyzed.


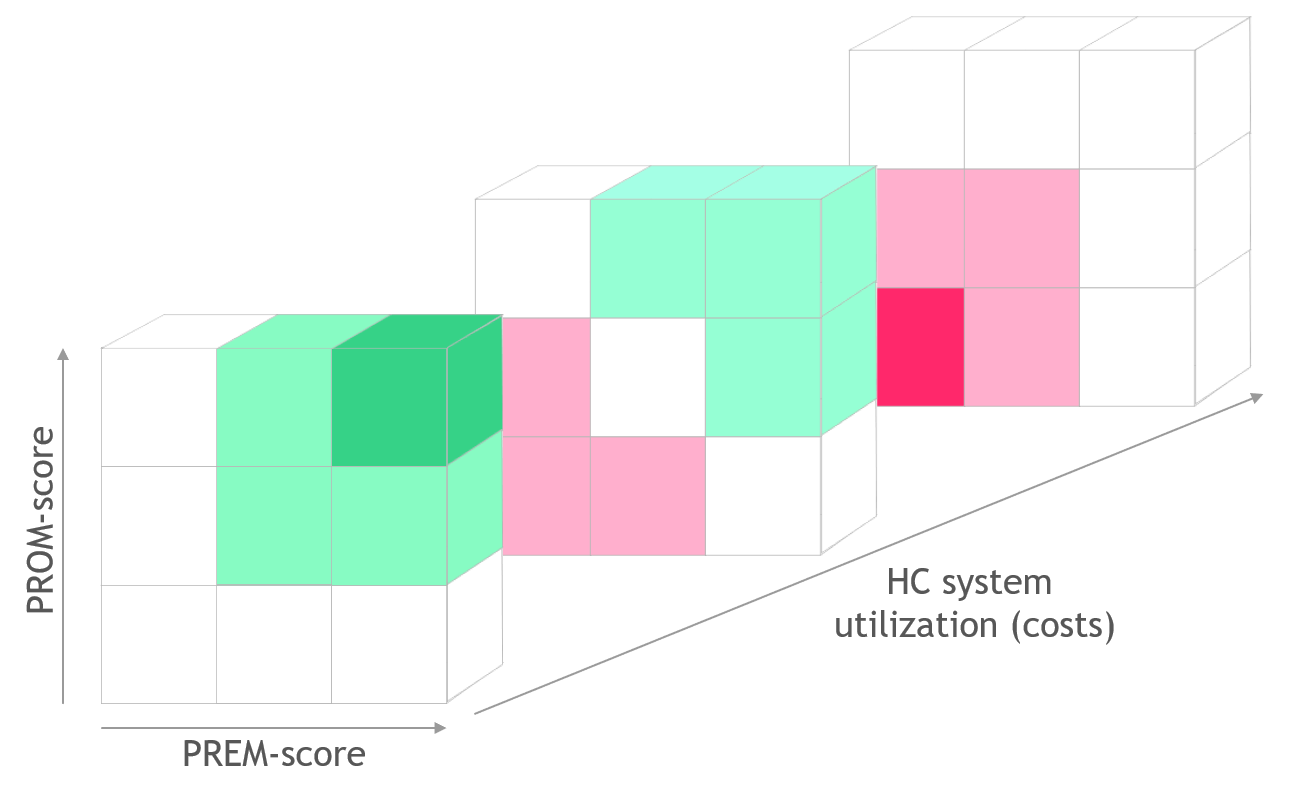


*Figure 6: Assessment of Value-Based Healthcare – Framework distinguishing between high- and low-value care*

*Outcome and experience variation*

Mean change in PROM and PREM-index (sub-)scores overall and per subgroup (see 5.1 and 4.3) per disease area over time:

- Average per survey period
- Consistency over time (test-retest reliability)

Meaningful change / Minimally clinical important difference (MCID) in PROM index (sub-)scores overall and per subgroup (see 5.1 and 4.3) per disease area over time:

- Average per survey period
- Consistency over time (test-retest reliability)

*Hospital admission risk based on PROMs/PREMs and patient characteristics*

- Using machine learning predictive models (long-short-term memory deep learning network for time trend classification, random forest, logistic regression, decision tree and/or support vector machine) to identify admission risk in Q3 and Q4 based on PROMs/PREMs in Q1 and Q2

*PRO feedback*

- Mainly: Statistically significant change in at least one of the health care behavior dimensions (see 4.2) and comparison of participants who opened the report vs. those who did not, using logistic regression (change/no change)
  - Physical activity: Shift of participants to a higher category
  - Nutrition: Shift of participants to “More healthy” from any other category
  - Tobacco: Shift of participants to a lower category
  - Alcohol: Shift of participants to a lower category
  - Sleep: Share of stable participants (no significant variation in sleep duration)
- Secondly: Descriptive exploratory analysis of the rates of the following two evaluation dimensions and statistically significant differences in subgroups (see 5.1 and 4.3):
  - Understandability
  - Helpfulness
  - Emotional change

**Data analysis**

- Check for normality using the Shapiro–Wilk test
- Check for intraclass correlation using intraclass correlation coefficients
- Analyze missing data
- Descriptive statistics of patient and treatment characteristics
- Time series analysis
- Parametric and non-parametric methods
- Statistical tests depending on normality for the primary and secondary outcomes to compare:
  - Responders vs. non-responders (to the nudge)
  - DMP vs non-DMP members
- Linear and logistic regressions controlling for:
  - Significant patient characteristics
  - Significant other collected variables depending on the research question
  1. SECONDARY DATA ANALYSES

**Utilization of healthcare services overall and per subgroups based on claims data**

- Outpatient care data: average frequency (days, cases) of outpatient practitioner visits by specialty, with average costs per case, visit, and overall costs per patient-year, pre- and post-intervention time.
- Prescriptions of medications: number of outpatient prescriptions of medications with average daily doses per therapeutic area (as per ATC classification), overall costs per patient-year, pre- and post-intervention.
- Allied health professions (podiatric, dietary, speech and language, occupational and physiotherapy): average amount/duration of prescribed remedies per diagnosis group, cardinal symptom and remedy type per patient-year, pre- and post-intervention.
- Assistive technology prescription: frequency of prescription of assistive and therapeutic appliances per product group and patient-year, pre- and post-intervention.
- Inpatient hospital stays: number, duration, average cost of inpatient hospital stays per leading diagnosis and patient-year, pre- and post-intervention.
- Outpatient hospital care: number, duration, average costs of outpatient hospital care per leading diagnosis and patient-year, pre- and post-intervention.
- Work incapacitations: number and duration of work incapacitation per ICD-10-GM main groups and patient-year, pre- and post-intervention.
- Transportation costs: frequency, duration, and type of transportation, average costs per patient-year, pre- and post-intervention
- Household assistance: frequency, duration, type of assistance, and average costs per patient-year, pre- and post-intervention
- Nursing care: frequency, duration, type of care, and average costs per patient-year, pre- and post-intervention
- Rehabilitation: frequency, duration, type of rehabilitation, and average costs per patient-year, pre- and post-intervention
- Long-term care benefits: highest level of care, frequency, duration, type of care, and average costs per patient-year, pre- and post-intervention
