# Supplementary material for: Evaluating the Population-Based Usage and Benefit of Digitally Collected Patient-Reported Outcomes and Experiences in Patients With Chronic Diseases: The PROMchronic Study Protocol
Source: JMIR Res Protoc. 2024 Aug 5;13:e56487. doi: 10.2196/56487 (PMC11333866; doi:10.2196/56487)

| BARMER · 42266 Wuppertal | 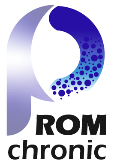  October 2023 |
| --- | --- |
| Mrs. |  |
| Maria Mustermann  Musterstr. 99  12345 Berlin |  |

Invitation to Participate in the Health Care Study "PROMchronic"

Dear Mrs. Mustermann,

with this letter, we would like to invite you to participate in the study **PROMchronic - The Potential of Patient-Reported Outcomes to Improve the Care of Chronically Ill Patients**. PROMchronic is conducted by the Technical University of Berlin, the Institute for Applied Quality Improvement and Research in Healthcare (aQua-Institut), and Oncare, in collaboration with your BARMER.

**Research Objective**: The aim of the study is to explore the use of questionnaires that capture **health, quality of life, and care situations from the patients' perspective**. This is currently rarely utilized in the German healthcare system. Hence, this research project aims to determine whether the questions used in the study can be employed and whether care deficits can be identified.

**Feedback on your information:** As a study participant, you will regularly receive insights into your own survey results compared to nationwide study participants. This way, if necessary, you can discuss and possibly adjust your care needs individually with your doctor. Additionally, by participating, you are supporting the evidence-based advancement of German healthcare design.

**How to Participate:** Please use your study pseudonym *XXX* for registration. You can participate in the study via the digital health app Myoncare by ONCARE GmbH or through a web browser on your mobile or desktop computer. All further information regarding direct participation, study procedures, and data protection can be found at: *individual link to survey*

**Or** by scanning the QR code using your smartphone's camera:

QR-code placeholder

Participation in the study is voluntary, and you will not experience any disadvantages if you choose not to participate. If you decide to join the study, you will receive quarterly questionnaires over a year. By participating, you are aiding research in the field of chronic diseases. The insights gained from this study aim to help enhance the care of chronically ill patients in the long run.

**Do you have any questions?** You can reach the study hotline on Mondays and Thursdays between 9 am and 1 pm at the phone number +49 30 314 77454 or via email at promchronic@mig.tu-berlin.de.

We sincerely thank you for your support!

Warm regards


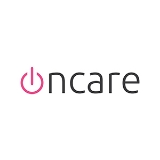
Your BARMER Our partners in healthcare

For the research consortium
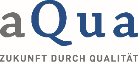

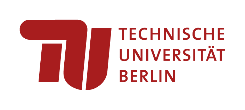

Supplement: Multimedia Appendix 2 [file resprot_v13i1e56487_app2.docx]
