# Supplementary material for: Evaluating the Population-Based Usage and Benefit of Digitally Collected Patient-Reported Outcomes and Experiences in Patients With Chronic Diseases: The PROMchronic Study Protocol
Source: JMIR Res Protoc. 2024 Aug 5;13:e56487. doi: 10.2196/56487 (PMC11333866; doi:10.2196/56487)
Supplement: Multimedia Appendix 3 [file resprot_v13i1e56487_app3.docx]

**Study Information for Patients**

**Version dated April 17, 2023**

| Research Project PROMchronic |
| --- |
| Sponsor/Commissioner: “Innovationsfonds des Gemeinsamen Bundesausschuss”  Consortium leadership: Technical University of Berlin - Department of Healthcare Management,  Faculty of Business & Management. *Straße des 17. Juni 135 10623 Berlin* |
|  |

**Dear study participants,**

We are pleased that you are interested in participating in the research project "*PROMchronic - Potential of PROMs and PREMs to improve the care of patients with chronic diseases*."

The project is funded by the “Innovationsfonds” for the promotion of new care forms (§ 92a Abs. 1 SGB V) (Funding nr.: 01VSF21037).

Responsible for the implementation of the project are the Technical University of Berlin (Department of Healthcare Management), the aQua – Institute for Applied Quality Promotion and Research in Healthcare GmbH, BARMER, and ONCARE GmbH.

# What is the goal of the study, and what are benefits of your participation?

In the PROMchronic research project, survey instruments are used to evaluate health, quality of life, and the care situation from the patients' perspective. Currently, in Germany, outcome assessments in healthcare do not include the patients' perspective. Therefore, the research project aims to determine whether the questions used in the study could be employed for this purpose. Additionally, the objective is to enhance the quality of treatment for patients with chronic diseases by comparing nationwide care. We are contacting you via the health insurer to include patients from different regions of Germany. Since the study is funded by a government organization (“Gemeinsamer Bundesausschuss”), the study's results will be made available to decision-makers in the German healthcare system for future consideration. Thus, by participating, you can help shape the evidence-based reality of care for tomorrow. The insights gained from this study aim to improve the care of patients with chronic diseases in the long run.

Within the context of the study, you can also benefit by receiving your own health outcomes compared to a reference group and adjusting your health and care behaviors accordingly.

# How does the research project proceed, and what is your involvement?

You will be asked to complete a questionnaire about your well-being and your experience with healthcare services using a digital device (tablet, mobile phone, computer) quarterly over a period of one year.

A prerequisite for participating in this study is the use of a personal account on the myoncare platform, which you should create using only the pseudonym provided to you. The IP address you use will not be processed or stored.

If you encounter any difficulties completing the questionnaires at any point, you are welcome to discuss them with your relatives or other trusted individuals, or you can contact the person mentioned in this document for assistance at any time.

# Is participation voluntary?

Your participation is voluntary. You can only be included in the study if you agree. If you choose not to participate, there will be no disadvantages for you. You have the right to revoke your given consent to participate in the study or the further processing of your data at any time until the end of the overall survey period and to terminate your participation in the study.

# What risks and efforts are involved for you as a participating patient?

There are no potential risks for you due to your inclusion in the study. Pseudonymized data will be collected, and no keys for re-pseudonymization will be shared. The key for re-pseudonymization remains in the possession of your health insurer BARMER throughout the entire duration of the study and even afterward (until the data is deleted). The pseudonymized data will be handled carefully and confidentially in accordance with the reviewed data protection concept. Your data will be processed anonymously and strictly for study purposes. Your effort involves completing the digital questionnaires sent to you.

# What are the reasons for discontinuing the study and how can participation be revoked?

The study will only be discontinued if you decide that you no longer wish to participate. If you wish to discontinue, you can register your withdrawal directly on the myoncare platform.

If you revoke your consent to the processing of data before the end of the collection period, the relevant data will be deleted, and no further data will be collected.

# What happens to your data after the survey period has ended?

To scientifically investigate the extent to which regular surveys of patients with chronic diseases can have an impact on healthcare, your data will be evaluated together with the data of other study participants. For this purpose, your data will be factually anonymized. This means that it is not possible for the researchers to draw any conclusions about your identity.

The pseudonymized data will be stored for analysis purposes at the research institutions (aQua-Institute and Department of Healthcare Management at TU Berlin). The analysis period is two years. To allow for verification of the study results after the project's completion, the pseudonymized data will be stored for a period of 10 years after the end of the project. At no point can any conclusions be drawn about your identity.

# What do we need your consent for?

For participation in PROMchronic, we require your consent. This consists of two declarations, which will be explained in the following.

- **Your Participation:** Your agreement to participate in PROMchronic is a prerequisite for contributing to the study. By doing so, you agree that the data you provide in the electronic questionnaire will be scientifically evaluated.
- **Your Health Insurance Data:** Additionally, you can agree that selected data stored about you at your health insurance company (e.g., information on prescribed medications) be included in the scientific evaluation.

# Who has access to my data and how is it protected?

The collection, storage, and processing of personal data in this project are in accordance with the provisions of the General Data Protection Regulation, the specific data protection provisions of the “Sozialgesetzbuch”, and all other national data protection regulations. The myoncare software of ONCARE GmbH is used for your answers to the electronic questionnaires. Data collection is exclusively pseudonymized, i.e., without allowing conclusions about your person. For scientific evaluation, your pseudonymized data are then passed on to the researchers.

# The PROMchronic study is conducted by experienced institutions

The Technical University of Berlin, **Department of Healthcare Management**, is a public corporation of the State of Berlin. The **aQua - Institute for Applied Quality Enhancement and Research in Healthcare GmbH** is a privately-owned service company rooted in the scientific setting. **BARMER**, as your insurer, is a public law corporation. **ONCARE GmbH** is a private service company certified by TÜV Süd.

- All institutions involved in the project strictly adhere to the provisions of the General Data Protection Regulation, the specific data protection provisions of the “Sozialgesetzbuch”, and all other national data protection laws. Therefore, the transfer of personal data to third parties is prohibited. Your data, adhering to data protection provisions, is exclusively used for scientific evaluation.
- Data analysis is solely carried out by the aQua Institute and the Technical University of Berlin.
- The analysis results are presented exclusively in anonymized form and aggregated by groups. This means that no one can later determine from the results which individual provided the information or which data corresponds to which person. The research project's findings will be published in medical literature exclusively in an anonymized form.
- Your consent declarations for participation in the study and the data processing are stored on ONCARE GmbH's servers in compliance with data protection provisions and will be destroyed at the end of the study.

**You can terminate your participation in the study at any time and object to the further processing of your data collected within the scope of the study. In such a case, please register your revocation directly in the myoncare application.**

The project has been coordinated with all the official data protection officers of the project partners. For inquiries concerning data protection within the project, please contact:

Technical University of Berlin, Prof. Dr. med. Reinhard Busse, Straße des 17. Juni 135 - Secretariat H80, 10623 Berlin, E-mail: [mig@t-berlin.de](mailto:mig@t-berlin.de)

# Whom can I contact with questions?

Should you have subsequent questions about the research project "PROMchronic - Potential of PROMs and PREMs to Improve the Care of Chronically Ill Patients," you can contact the following person at any time using the provided contact details:

| CONTACT OF TU BERLIN |
| --- |
| Mr. Janis Nikkhah |
| Straße des 17. Juni 135 – Secretariat H80 |
| 10623 Berlin |
| Phone +49 (0)30-314-26933 |
| [janis.nikkhah@tu-berlin.de](mailto:janis.nikkhah@tu-berlin.de) |

**Right to lodge a complaint with the supervisory authority** (Berliner Commissioner for Data Protection and Freedom of Information, Friedrichstraße 219, 10969 Berlin, E-Mail: [mailbox@datenschutz-berlin.de](mailto:mailbox@datenschutz-berlin.de))

**Information on the right to access (Art. 15)**

You have the right to obtain information about the data we process about you.

**Information on the right to rectification (Art. 16) and/or data erasure (Art. 17)**

Should the data concerning you be incorrect or incomplete, you can request the correction of inaccurate data or the completion of incomplete data. You can request the deletion of your data at any time.

**Information on the right to restrict processing (Art. 18)**

You have the right to request a restriction on the processing of your personal data.

**Information on the right to data portability (Art. 20)**

Upon request, the files stored about you will be provided in a structured, commonly used, and computer-readable format.

**Informed Consent**

For participation in the scientific study: "PROMchronic - Potential of PROMs and PREMs to Improve the Care of Chronically Ill Patients" by the project partners Technical University of Berlin, aQua Institute for Applied Quality Promotion and Research in Healthcare GmbH, BARMER, and ONCARE GmbH. The declaration of study participation, as well as the consent for processing routine health insurance data, are requested and recorded on the welcome page of the digital survey (see following).

**PATIENT – Study Participation**

By giving your consent, you agree that your survey data will be collected, stored, and processed in a pseudonymized form for the purpose of conducting the study intervention and for research purposes at the aQua Institute and the TU Berlin. Furthermore, you consent to be contacted via email and through the myoncare application.

I am aware that I have the right to withdraw my **consent at any time up to the conclusion of the last survey without specifying reasons and without any adverse consequences for me** and can object to further processing of my data and request its deletion.

**I declare that I voluntarily agree to participate in the “PROMchronic” study**.

◻ Yes *[please tick here if applicable]*

**PATIENTS – Processing of Routine Health Insurance Data**

In addition to participating in the study, you have the option to give your consent for the pseudonymized collection, processing, and personal linkage of routine data from the BARMER statutory health insurance.

By giving your consent, you agree that your health insurance data will be linked with your survey data. This pertains exclusively to medical data and details about your utilization of health care services. By consenting to the processing of health insurance data, you enable analyses in health care research by incorporating pseudonymized service data.

**I agree that the project-related routine data from my BARMER health insurance will be collected, pseudonymized, processed, and linked for research purposes:**

◻ Yes ◻ No
