# Supplementary material for: Evaluating the Population-Based Usage and Benefit of Digitally Collected Patient-Reported Outcomes and Experiences in Patients With Chronic Diseases: The PROMchronic Study Protocol
Source: JMIR Res Protoc. 2024 Aug 5;13:e56487. doi: 10.2196/56487 (PMC11333866; doi:10.2196/56487)
Supplement: Multimedia Appendix 4 [file resprot_v13i1e56487_app4.pdf]

IndikationAsthma

Study pseudonym: [123456]

Diagnosis: Asthma

Date of Birth: [DD.MM.YYYY]

Gender: [Female]

promchronic@mig.tu-berlin.de

(030) / 314 77454

Monday, Thursday: 09:00 - 13:00 Uhr

Your personal Health Report

Report created on [20.09.2022]

—

Please find enclosed your health-related results compared to a comparison group similar to you. Your comparison group is made up of [XXX] participants in this [first] survey period and has the following characteristics:

Gender: [Female], Age: [20-26], Study group: Asthma.

If you have participated in more than one survey, you will also see your values over time.

General Health Status

- Your values
- Average values of your comparison group

Higher value = better Max Value: 100

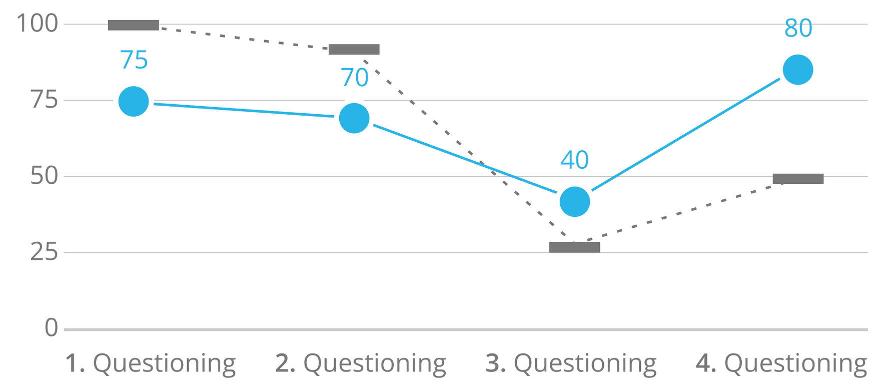

Lower value = worse

Health Status related to Asthma

- Your values
- Average values of your comparison group

Higher value = better Max Value: 100

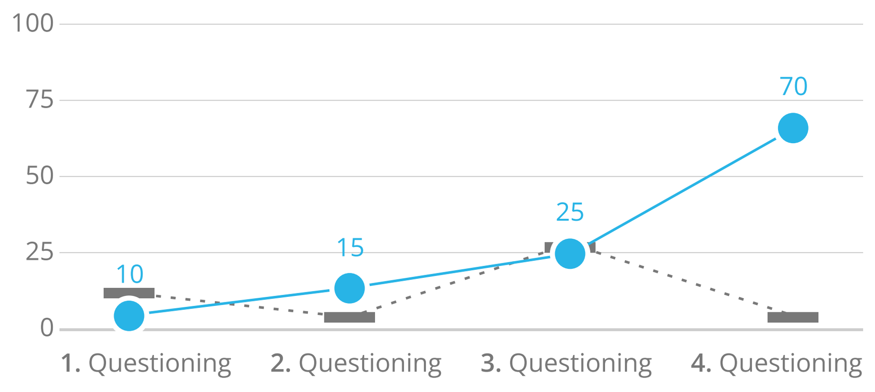

Lower value = worse

Explanation of your values

If your score (blue dot) is higher than the score of the comparison group (grey line), this indicates a better health status.

Even if their results are worse than the average value of their comparison group, you can still do well. Your first point of contact for questions about your health and the classification of your values is your attending doctor.

On the next page you will find additional information about your health survey.

Information on the measurement of general health status

The general questioning is mapped by means of a validated questionnaire (PROMIS PROPr). This value indicates the general state of health. The following seven sub-areas are taken into account when calculating this value: Cognitive functions, depressiveness, fatigue, impairment due to pain, physical functioning, sleep impairment, participation in social roles and activities.

Information on the measurement of health status related to asthma

The AIRQ® is designed to help your doctor talk to you about your asthma control. The AIRQ® does not diagnose asthma. Regardless of your AIRQ® result (total number of YES answers), it is important that your doctor discusses the number and your answers to each question with you. All patients with asthma, even those who are well controlled, can have an asthma attack. The worse your asthma control becomes, the higher the risk of an asthma attack. Your doctor can decide how best to assess and treat your asthma.

Information on the standardisation of the results

For a better graphical illustration and easier readability, the results of the questionnaires were standardised on a scale from 0 to 100. The value 0 indicates the worst possible result and the value 100 the best possible result.
